# Chitosan-Based Nanomedicine to Fight Genital *Candida* Infections: Chitosomes

**DOI:** 10.3390/md15030064

**Published:** 2017-03-04

**Authors:** Toril Andersen, Ekaterina Mishchenko, Gøril Eide Flaten, Johanna U. Ericson Sollid, Sofia Mattsson, Ingunn Tho, Nataša Škalko-Basnet

**Affiliations:** 1 Drug Transport and Delivery Research Group, Department of Pharmacy, Faculty of Health Sciences, University of Tromsø The Arctic University of Norway, 9037 Tromsø, Norway; toril_andersen@hotmail.com (T.A.); goril.flaten@uit.no (G.E.F.); 2 Host Microbe Interactions Research Group, Department of Medical Biology, Faculty of Health Sciences, University of Tromsø The Arctic University of Norway, 9037 Tromsø, Norway; ekaterina.mishchenko@uit.no (E.M.); johanna.e.sollid@uit.no (J.U.E.S.); 3 Department of Pharmacology and Clinical Neuroscience, Division of Clinical Pharmacology, Umeå University, SE-90187 Umeå, Sweden; sofia.mattsson@pharm.umu.se; 4 Personalized Dosage Form Design Research Group, School of Pharmacy, Faculty of Mathematics and Natural Sciences, University of Oslo, 0316 Oslo, Norway; ingunn.tho@farmasi.uio.no

**Keywords:** chitosan, liposomes, drug delivery, vaginal therapy, *Candida albicans*, metronidazole

## Abstract

Vaginal infections are associated with high recurrence, which is often due to a lack of efficient treatment of complex vaginal infections comprised of several types of pathogens, especially fungi and bacteria. Chitosan, a mucoadhesive polymer with known antifungal effect, could offer a great improvement in vaginal therapy; the chitosan-based nanosystem could both provide antifungal effects and simultaneously deliver antibacterial drugs. We prepared chitosan-containing liposomes, chitosomes, where chitosan is both embedded in liposomes and surface-available as a coating layer. For antimicrobial activity, we entrapped metronidazole as a model drug. To prove that mucoadhesivness alone is not sufficient for successful delivery, we used Carbopol-containing liposomes as a control. All vesicles were characterized for their size, zeta potential, entrapment efficiency, and in vitro drug release. Chitosan-containing liposomes were able to assure the prolonged release of metronidazole. Their antifungal activity was evaluated in a *C. albicans* model; chitosan-containing liposomes exhibited a potent ability to inhibit the growth of *C. albicans*. The presence of chitosan was crucial for the system’s antifungal activity. The antifungal efficacy of chitosomes combined with antibacterial potential of the entrapped metronidazole could offer improved efficacy in the treatment of mixed/complex vaginal infections.

## 1. Introduction

Vaginal infections are extremely prevalent and are reported to be one of the most common reasons for women to seek professional healthcare. Surveys estimate that more than 70% of adult women have experienced vaginal problems and have used vaginal products to treat infections [1]. The most common among vaginal infections are bacterial vaginosis, trichomoniasis, and vulvovaginal candidiasis [2,3,4]. In addition to causing discomfort and affecting the quality of life of infected individuals, these infections are also associated with an increased risk of related complications. There is an increased susceptibility to other pathogens such as human immunodeficiency virus (HIV), *Herpes simplex* virus, Neisseria *gonorrhoeae*, and *Chlamydia trachomatis*. Among pregnant women, there is a risk of preterm labor and birth and late fetal loss [5,6]. Current treatment regimens for these vaginal infections are considered to be insufficient due to a high degree of recurrence and limited patient compliance. The emergence of antimicrobial resistance to chemotherapeutics and the management of recurrent infections puts strong emphasis on the need for more efficient local therapies. Local treatment of vaginal infections has become favored over oral drug administration because, if properly applied, it can assure a higher local drug concentration and reduce the incidence of drug interaction, as well as interference with the gastrointestinal tract [1]. In past years, more attention has been given to the importance of biofilms and the treatment failure due to the inability of the selected drug to disrupt biofilms leading to bacterial survival and the recurrence of infection [7]. In spite of the increased interest to address the need for superior local treatment, most of the conventional dosage forms applied to the vagina are still suffering from poor distribution and retention at the vaginal site [8]. Moreover, the causes of vaginal infections might be often both of bacterial and fungal origin [9]. We aimed to develop an advanced mucoadhesive delivery system based on the synergistic antimicrobial action of a commonly prescribed drug to treat bacterial vaginosis, metronidazole [10], incorporated in chitosan-based mucoadhesive vesicles expected to exhibit antifungal activity on their own.

Chitosan is a natural polysaccharide obtained by the deacetylation of chitin. It is biocompatible, biodegradable, and non-toxic and thereby an interesting substance as a pharmaceutical excipient [11,12]. Chitosan has demonstrated excellent mucoadhesive properties and is therefore a good candidate for incorporation in dosage forms and delivery systems targeting the vaginal site; chitosan is expected to assure a prolonged vaginal residence time of the drug dosage form/delivery system [13,14,15]. In addition to its wide use in conventional dosage forms, chitosan has also been extensively studied as a constituent in nanosystems, such as chitosan-based nanoparticles or nanoemulsions, or as a coating material for liposomes [16,17,18,19,20,21,22]. We were particularly interested in the fact that chitosan exhibits potent antimicrobial, particularly antifungal, effects [23,24,25]. Chitosan was able to disrupt the biofilm integrity and exhibited antifungal activity against *Candida* species [26] or bactericidal effects against *Pseudomonas aeruginosa*. Importantly, the biofilm disruption was not depended on the simulated pH conditions; it was slightly more potent at the less acidic pH [27].

To prove the superiority of chitosan-based delivery systems in antimicrobial vaginal therapy, we used the mucoadhesive polymer Carbopol as a control. Carbopol is widely used in pharmaceutical applications as a constituent in different mucoadhesive drug delivery systems [8], including the ophthalmic, nasal, buccal, topical, intestinal, and vaginal therapy sites [28,29], due to its accessibility, stability, and safety. In vaginal drug delivery, Carbopol is often studied as a constituent in bioadhesive gels [30].

To prepare novel mucoadhesive formulations with either chitosan or Carbopol, we applied our own one-pot preparation method for polymer-containing liposomes [14,31]. This preparation method results in a polymer coating at the surface of the liposomes as well as a proportion of the polymer coating being inside the aqueous compartments of the liposomes and ensures sufficient drug loading. To prove the antifungal activity of chitosan and the ability of chitosan-containing liposomes to successfully carry antimicrobial drugs, we prepared chitosan-containing liposomes loaded with metronidazole, a known antimicrobial drug without intrinsic antifungal potential against *Candida albicans*. The activity was compared to the control, Carbopol-containing liposomes, as well as plain liposomes, both loaded with metronidazole, at the pH conditions mimicking the infected vagina (neutral pH) [4].

## 2. Results and Discussion

Chitosan-based nanomedicine for the localized therapy of vaginal infections should be characterized by optimal vesicular size, surface properties, and drug load, as well as the ability to assure the prolonged release of the incorporated drug within the vaginal cavity.

### 2.1. Characterization of Vesicles

The size of non-sonicated chitosan-containing vesicles is known to be greater than 1 micron, exhibiting a high polydispersity (expressed as high polydispersity index, PI) [31]. To reduce the polydispersity and vesicle size, the parameters that affect the retention time, the release of incorporated drug, and consequently its efficacy, sonication was applied and its duration optimized to prepare vesicles of a suitable size (100–300 nm), considering targeted vaginal administration [22]. Smaller more uniformly sized vesicles are expected to have a better distribution in the vaginal cavity and to penetrate deeper into the mucosal layer [32]. Deeper penetration will improve the residence time as the vesicles could penetrate through the rapidly clearing upper layer of the mucus, thereby improving the treatment efficacy [33]. The prepared vesicles fitted to a bimodal size distribution (NICOMP) and the volume-weighted percentages of particles in each population are presented in Table 1. All the vesicles were initially sonicated for 1 min. As previously reported, the chitosan-containing liposomes were smaller than the plain liposomes after the size reduction [14,31]. The Carbopol-containing liposomes seemed to resist size reduction to a greater extent than the other types of liposomes. After 1 min of sonication, the size of the Carbopol-containing liposomes was only reduced to 500 nm, as opposed to around 200 nm for both the chitosan-containing and the plain liposomes (Table 1). The Carbopol-containing liposomes were therefore subjected to another 1 min of sonication and the size was determined again. The size of the vesicles was this time reduced to 400 nm and remained larger than other vesicles. It seems that Carbopol, at the concentrations used, has a stabilizing effect on the polymer-containing liposomal structure; the same stabilizing effect was not observed for chitosan.

The surface properties of the vesicles were characterized through the zeta potential of the different formulations and are presented in Table 2. Plain liposomes exhibited a neutral zeta potential as was expected for phosphatidylcholine (PC) liposomes, which is a neutral lipid. The chitosan-containing liposomes exhibited a positive zeta potential due to the presence of positively charged chitosan on the surface, in agreement with our previous work. However, the values were slightly higher than previously reported [14], which can be attributed to the batch to batch differences in chitosan properties. The differences were not significant. The Carbopol-containing liposomes of two different sizes had a negative zeta potential, although not pronounced. This could be a reflection of both the amount of Carbopol at the surface of the liposomes and the degree of ionization of the carboxylic acids of Carbopol. Carbopols have a pKa of 6 [34] and the pH of the solution was 4.9; therefore the polymer would not be fully ionized. An additional explanation could also be that some of the Carbopol was embedded within the liposomal bilayers, as we have previously reported for chitosan in chitosomes [14]. This hypothesis might be supported by the published data on Carbopol-coated liposomes (post-coating of formed liposomes), wherein no Carbopol could be found embedded within liposomal bilayers [20]; the authors reported a zeta potential of −10.5 mV at the concentration of the coating solution similar to that used in current study. The differences in the expected and measured zeta potential for Carbopol-coated and our Carbopol-containing liposomes confirm that the preparation method rather than the type of polymer used determines the polymer distribution within/onto the vesicles.

### 2.2. Metronidazole Entrapment Efficiency

A key aspect of successful treatment of local vaginal infections is assuring that a sufficient amount of the drug is readily available at the vaginal site for a sufficient period of time [35]. Localized metronidazole therapy is a safe alternative to oral metronidazole treatment; vaginal metronidazole gel exhibited a 96% reduction in total systemic exposure [36], which is of great importance in the therapy of pregnant patients.

**Figure 1:**
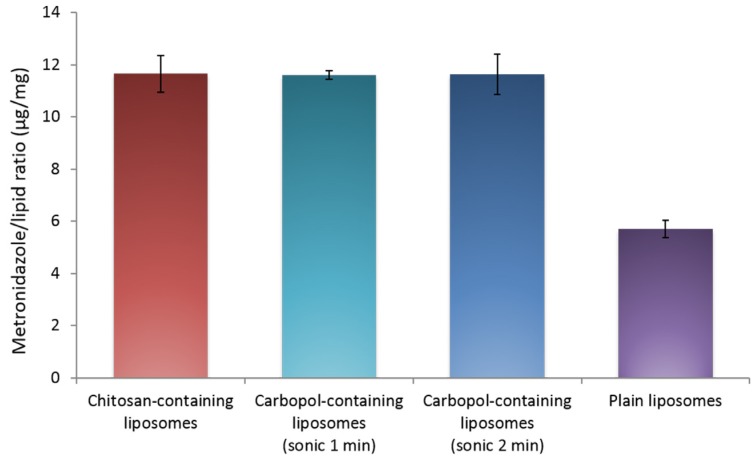
Entrapment efficiency of metronidazole in chitosan-containing liposomes, Carbopol-containing liposomes, and plain liposomes. All liposomes were sonicated for one minute, unless stated differently. All values represent the mean ± SD (*n* = 3).

Figure 1 represents the entrapment efficiency of the different liposomal formulations, expressed as the amount of metronidazole per mg of liposomal lipid, normalized after the accurately determined amount of lipid in each formulation. Smaller and larger Carbopol-containing liposomes are presented as Carbopol-containing liposomes sonicated for one and two minutes, respectively.

As previously shown [31], the plain liposomes entrapped the lowest amount of metronidazole at about 6 μg/mg lipid, which is significantly less than the polymer-containing formulations (*p* < 0.001). The chitosan-containing liposomes and both of the Carbopol-containing liposomes were characterized by the entrapment efficiency of 11–12 μg/mg lipid and were not significantly different from each other. This indicates that the presence of polymer, rather than the type of polymer used, determines the entrapment efficacy. Chitosan-containing liposomes in the current study (1 min sonication) had a higher entrapment efficacy than in our previous work [31]. The finding can be attributed to a change/optimization in the sonication method; in the previous study [31] the vesicles were not diluted prior to sonication. The sonication of the more concentrated suspensions could lead to the higher loss of the entrapped drug, resulting in a lower final drug load.

### 2.3. In Vitro Release Studies

Nanomedicine has great potential in the development of superior delivery systems to treat vaginal diseases locally [37]. Metronidazole is an antimicrobial with a high potential for local administration against anaerobic bacteria and protozoa pathogens [38]; its importance in the local treatment of vaginal infections is decades long and remains attractive.

**Figure 2:**
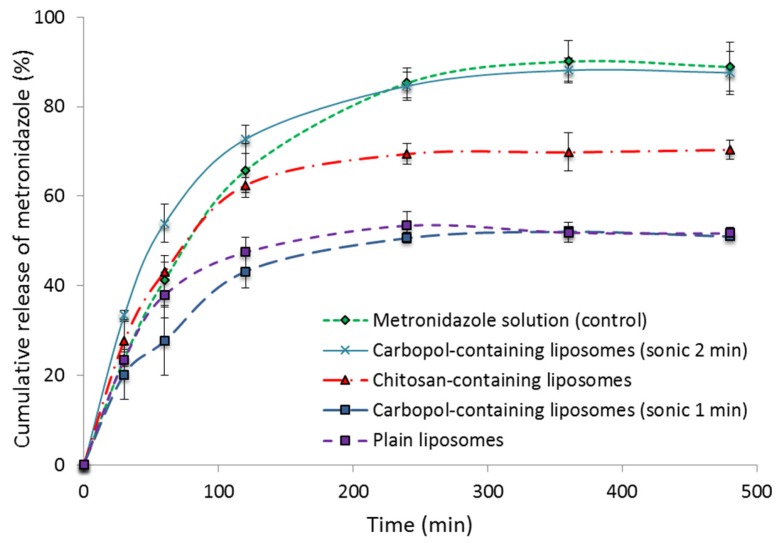
Cumulative release of metronidazole from different types of liposomes at pH 7. All liposomes were sonicated for one minute unless stated differently. The values represent the mean ± SD (*n* = 3).

Figure 2 shows the cumulative release of metronidazole from the different types of vesicles containing metronidazole; metronidazole in solution served as a control. The testing was performed at neutral pH, mimicking the conditions of an infected vagina. Based on our previous work with chitosan-coated vesicles, we expected that the mucoadhesiveness of the chitosomes would not be affected by the pH [39]. All the polymer-containing liposomes sonicated for one minute exhibited a sustained release of metronidazole compared to the control solution. The Carbopol-containing liposomes, sonicated for longer time (2 min), exhibited a release that did not differ significantly from the control (metronidazole in solution), failing to assure a sustained drug release. The explanation of why Carbopol-containing vesicles of different sizes exhibit different drug release profiles might be that the drug was more loosely associated with smaller Carbopol-containing vesicles and was released faster from these than from larger vesicles of the same type (sonicated for 1 min). Figure 1 indicates that both smaller and larger Carbopol-containing vesicles entrapped a similar amount of metronidazole; the difference was in their size and surface charge (Table 2). It seems that Carbopol as a polymer can protect a vesicular structure from forced size reduction to a certain degree; when less Carbopol becomes surface available after longer sonication (closer to neutral zeta potential, Table 2), the vesicles become leakier. The phenomenon needs to be further evaluated, and, at this stage, we can only postulate on the reasons behind the observations and the difference in packing within vesicle bilayers between chitosomes and Carbopol-containing vesicles. However, the Carbopol-containing vesicles only served as a control in the antimicrobial testing; we did not invest time in further exploration of this interesting observation.

The Carbopol-containing liposomes, sonicated for 1 min, sustained the delivery of metronidazole the most, similarly to plain liposomes (Figure 2). The finding could be attributed to their size and multilamelarity as compared to smaller vesicles which were probably oligolamellar. Chitosan-containing liposomes release drug in a more sustainable manner. We expected the drug to be completely released from the vesicles without reaching a plateau, as in the case of the metronidazole solution. Considering that the equilibrium reached between the drug associated with the vesicles in the donor chamber and the released drug in the acceptor chamber can slow the concentration gradient and contribute to the plateau and that different vesicles tend to accumulate on the membrane surface to a different extent, we cannot exclude the interaction between the membrane and the vesicles. However, considering the normal vaginal clearance and expected residence time within vagina [40], the drug release patterns for all three liposomal formulations are acceptable.

### 2.4. Antifungal Activity

Chitosan has been previously confirmed to exhibit high activity against *C. albicans* and to act on the prevention and disruption of biofilms of *C. albicans* [25,41,42,43]. It has also been reported that *C. albicans* biofilms are resistant to commonly used antifungal agents such as fluconazole [44,45]; therefore we expected our chitosan-containing liposomes to act on *C. albicans* inhibition and express potent antifungal activity. This would enable us to apply a synergistic approach in the therapy of vaginal infections, namely a carrier system with antifungal potential bearing a drug with antibacterial potential. This synergistic approach would combat the mixed infections and possibly act on antimicrobial resistance. Moreover, empty, drug free, chitosan-containing liposomes could be applied as a safe and effective antifungal treatment, which avoids exposure to drugs, which is particularly important in the treatment of pregnant patients [40].

As a first step we wanted to prove that chitosan-containing vesicles prevent the growth of *C. albicans* and that the presence of the drug within liposomes does not interfere with the activity. Therefore, the antifungal activity of the polymer-containing liposomes was tested by challenging the growth of *C. albicans* in the presence of the liposomal formulations during a 24 h-period. The chitosan-containing liposomes, both loaded with metronidazole as well as empty liposomes, exhibited a potent ability to inhibit the growth of *C. albicans* (Table 3). The lowest concentration of antifungal activity varied between 0.22 and 0.11 mg/mL of chitosan. The content of metronidazole in the drug-containing samples, 18 to 36 μg/mL, for chitosan- and Carbopol-containing vesicles respectively, did neither significantly alter nor improve the *C. albicans* inhibition compared to the non-drug containing (empty) liposomes.

For the Carbopol-containing liposomes and plain liposomes, as well as the metronidazole control solution, no inhibition of *C. albicans* growth was observed. This is expected since metronidazole has no known activity against *C. albicans*. The results are highly encouraging considering our hypothesis. Vaginal infections can be complicated and result in infection with multiple pathogens, both of bacterial and fungal origin, which may occur either simultaneously or successively, leading to a high degree of recurrence associated with mixed infections [9,46]. The addition of an antibacterial agent, such as metronidazole, to a drug delivery system that is potently able to inhibit fungal growth would be greatly beneficial for the overall treatment and prevention of recurrence. In addition, chitosan has demonstrated the ability to disrupt bacterial biofilms involved in bacterial vaginosis [27]. This confirms chitosan as a superior component in our vaginal drug delivery system since chitosan is expected to disrupt the biofilm allowing the vesicle-associated antibacterial agent to reach the bacteria that would otherwise be shielded by the biofilm. Importantly, chitosan has also been reported to act against *Trihomonas vaginalis* infections, further strengthening its potential in antimicrobial vaginal therapy [47].

Metronidazole is a good choice among antibacterial agents since many vaginal infections are susceptible to metronidazole [4]. In the treatment of metronidazole-sensitive vaginal infections, such as bacterial vaginosis and trichomoniasis, there is an additional advantage. While many antimicrobial agents have a damaging effect on the natural microflora, metronidazole has been demonstrated not to affect the *Lactobacillus* strains in the vagina. This leaves the flora able to maintain the beneficial pH in the vaginal environment, protecting against further infection from opportunistic pathogens [48].

Considering chitosan’s safety profile, mucoadhesiveness, and potent antimicrobial potential, chitosan can be considered a crucial constituent of novel nanosystems for vaginal administration. Moreover, its wound healing activities could assist in the treatment of vaginal lesions, which might be the pre-condition of vaginal infections.

## 3. Materials and Methods

### 3.1. Materials

Soy phosphatidylcholine (SPC; Lipoid S100, Lipoid GmbH, Ludwigshafen, Germany) was a generous gift from Lipoid GmbH. Chitosan (77% degree of deacetylation (DD), Fiske-SubbaRow reducer reagent, metronidazole, methanol, *n*-propanol, phosphorus standard, and Triton X solution were purchased from Sigma Aldrich Inc. (St. Luis, MO, USA). Ammonium molybdate and peroxide were purchased from Merck KGaA (Darmstadt, Germany), while sulfuric acid was purchased from May and Baker LTD (Dagenham, UK). Potato dextrose broth was purchased from Difco (BD, Franklin Lakes, NJ, USA) All other chemicals used in the experiments were of analytical grade. Carbopol^®^974P NF was a product from Lubrizol, Billingham, UK.

### 3.2. Preparation of Vesicles

**Figure A1:**
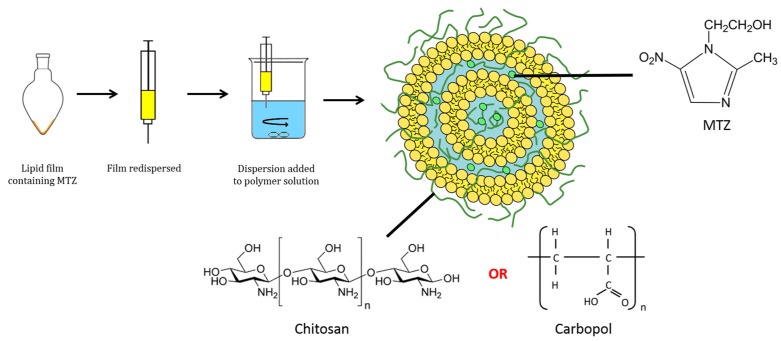
Schematic presentation of the preparation method.

Polymer-containing liposomes were prepared by the one-pot method previously described by Andersen et al. [31] (Appendix A Figure A1). In brief, SPC (200 mg) for the drug-free liposomes and SPC (200 mg) and 20 mg of metronidazole for metronidazole-containing liposomes were dissolved in methanol. The solvent was evaporated using a rotoevaporator system (Büchi rotavapor R-124 with vacuum controller B-721, Büchi Vac V-500, Büchi Labortechnik, Flawil, Switzerland) under a vacuum at 45 °C. The resulting lipid film was redispersed in 100 μL of *n*-propanol with a micro syringe pipette (Hamilton Company, Bonaduz, Switzerland). The dispersion was injected via a needle into 2 mL of aqueous solution of chitosan (0.17%, *w*/*w*, in 0.1% acetic acid), or Carbopol (0.10%, *w*/*w* in distilled water), and stirred for 2 h at room temperature. The dispersions were left in a refrigerator (4–8 °C) overnight prior to vesicle size reduction and characterization.

Plain (polymer-free) liposomes were prepared under the same conditions using the same lipid and metronidazole ratio to prepare the film. The film was subsequently re-dispersed (100 μL *n*-propanol), injected into distilled water, and stirred on a magnetic stirrer for 2 h. The dispersions were left in a refrigerator (4–8 °C) overnight prior to vesicle size reduction and characterization.

### 3.3. Size Reduction of Vesicles

The polymer-containing and plain (non-coated) liposomes were reduced to the desired size by sonication using a Sonics High Ultrasonic Processor (Sigma Aldrich Chemie GmbH, Steinheim, Germany). Prior to sonication, the samples were diluted to a suitable volume (5 mL) with distilled water. The duration of sonication was adjusted to achieve the desired size range of the vesicles (1 min, except for Carbopol-containing liposomes, which were also sonicated for 2 × 1 min). An ice bath was used to prevent heating of the samples.

### 3.4. Entrapment of Metronidazole

To remove unentrapped metronidazole from the polymer-containing, as well as the plain vesicles, the vesicles were dialyzed (Mw cut off: 12,000–14,000 Daltons; Medicell International Ltd., London, UK) against distilled water for 4 h at room temperature. The volume was adjusted to assure the sink conditions.

The amount of metronidazole entrapped in the vesicles was determined by UV-spectrophotometry (Agilent Technologies, Santa Clara, CA, USA). The samples were dissolved in methanol and metronidazole concentrations measured at 311 nm. The standard curve of metronidazole in methanol was prepared using the concentrations in the range of 2 to 20 μg/mL (*R*^2^ = 0.9999).

### 3.5. Lipid Content

The content of phosphatidylcholine (PC) in the vesicles was measured using the modified Bartlett method [49]. In brief, the samples were diluted by distilled water to appropriate concentration and an aliquot (1 mL) was mixed with 0.5 mL of 10 N H_2_SO_4_ and heated at 160 °C for a minimum of 3 h. After cooling, two drops of H_2_O_2_ were added, and the mixture was heated at 160 °C for 1.5 h. Then ammonium molybdate (4.6 mL; 0.22% *w*/*v*) and 0.2 mL of Fiske-SubbaRow reducer reagent were added after cooling, the samples were vortexed, and the mixture was heated for 7 min at 100 °C. All samples were analyzed by UV spectrophotometry at 830 nm. The phosphorus standard solution was used to prepare a standard curve in appropriate concentrations.

### 3.6. Particle Size Analysis

The size distributions of the sonicated vesicles were measured by photon correlations spectroscopy using a Submicron Particle-sizer (Model 370, Nicomp, Santa Barbara, CA, USA), according to the method described earlier [14]. Briefly, the samples were diluted with filtered distilled water (0.2 μm Millipore filters) to provide the appropriate count intensity (approx. 250–350 kHz) and measured in three parallels (runtime 10 min at 23 °C). Both Gaussian and Nicomp algorithms were fitted to the experimental data to find the distribution that best describes the particle population. The volume-weighted distribution was used to determine the mean diameter and polydispersity index (PI) of all samples.

### 3.7. Determination of Zeta Potential

The zeta potential of all vesicles was measured on a Malvern Zetasizer Nano ZS (Malvern Instruments Ltd., Oxford, UK). The instrument was calibrated throughout the measurements using the Malvern zeta potential transfer standard (−42 ± 4.2 mV). The samples were diluted in filtered water until an appropriate count rate was achieved and measured in a disposable folded capillary cell. All measurements were performed at 23 °C [22]. The results represent an average of at least three independent measurements.

### 3.8. In Vitro Release Studies

Release studies were performed using Franz diffusion cells (PermeGear, Hellertown, PA, USA), with the heating circulator (Julabo Labortechnik F12-ED, Seelback, Germany) maintaining the temperature at 37 °C and a neutral pH to mimic the pH of the bacterial vaginosis infected vagina [4]. Cells with 12 mL volume acceptor chambers and a diffusion area of 1.77 cm^2^ were used [14]. Polyamide membranes (0.2 μm pore size; Sartorius polyamide membrane; Sartorius AG, Göttingen, Germany) were used. The formulations were added to the donor compartment with a volume of 600 μL. The acceptor chambers were filled with distilled water and kept at 37 °C. Samples (500 μL) from the acceptor chamber were taken at 30, 60, 120, 240, 360, and 480 min and replaced with fresh medium. Both the sampling port and the donor chamber were covered with quadruple layers of para-film to prevent evaporation. Quantification of the released model substance was determined by spectrophotometry, wherein the aqueous solution of metronidazole was measured at 319 nm. The standard curve of metronidazole in distilled water was prepared using concentrations in the range of 2 to 20 μg/mL (*R*^2^ = 0.9988). All experiments were carried out in triplicate.

### 3.9. Antifungal Activity Testing

**Figure 3:**
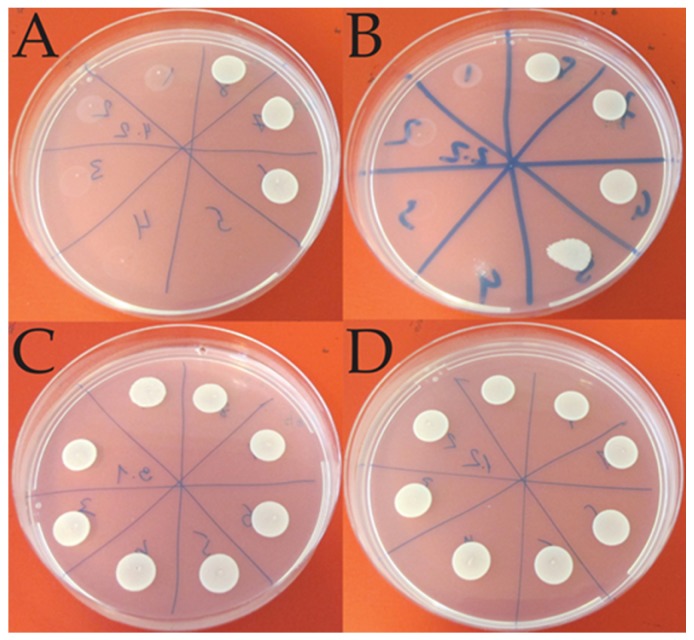
Representative photographs of the *C. albicans* growth on agar plates in the presence of different liposomal formulations; (**A**) Chitosan-containing liposomes (no drug); (**B**) Chitosan-containing liposomes (MTZ); (**C**) Carbopol-containing liposomes (MTZ); (**D**) plain liposomes (MTZ). Each sector contains an aliquot from a test well with a dilution of a formulation; aliquots were inoculated anticlockwise, i.e., from the highest concentration in sector 1 to the lowest concentration in sector 8. White spots represent the ‘lawn’ growth of *C. albicans*. The inhibition of *C. albicans* growth is indicated by the absence of white spots.

Antifungal activity was tested according to the method described by Sperstad et al. [50] with modifications, using the yeast strain *C. albicans* (ATCC 10231). Fungal cells were suspended in the potato dextrose broth (Difco, Sparks, NV, USA) with 2% glucose; the cell concentration was determined and adjusted after counting in a Bürker chamber. Aliquots (50 μL) of fungal cells (final concentration approx. 2 × 10^5^ cells/mL) were inoculated in a 96-well microtitre plate (Nunc^TM^, Roskilde, Denmark) along with 50 μL of vesicular formulation dissolved in Milli-Q water. The vesicular formulations were diluted in a two-fold sequence and tested at final concentrations ranging from 2.3 to 300 μg/mL. Cultures were grown in a dark chamber, without shaking, for 24 h at 37 °C. The growth inhibition was determined by plating aliquots of the samples on potato dextrose agar plates with 2% glucose (Figure 3) and was defined as the concentration at which no growth was observed after 24 h incubation at 37 °. In addition, the negative controls containing neither *C. albicans* nor vesicles and the controls containing only vesicles were also tested for growth.

### 3.10. Statistical Analysis

Students’ *t*-test was used for comparison of two means. A significance level of *p* < 0.05 was considered acceptable.

## 4. Conclusions

We developed chitosomes, chitosan-containing liposomes, of optimal size and sufficient metronidazole load for the localized therapy of mixed vaginal infections and have proven in vitro that this novel nanosystem can act on *C. albicans.* The chitosan-containing liposomes inhibited the growth of *C. albicans* independently of the presence of the entrapped model antibacterial drug, metronidazole, whereas the Carbopol-containing vesicles failed to prevent *C. albicans* growth. We have also shown that chitosomes provide a sustained release of the model drug, making it suitable for the administration of antibacterial drugs assuring the synergistic treatment of vaginal infections caused by several pathogens.

## Figures and Tables

**Table 1:** Size distribution of liposomes. All values represent the mean size and are volume-weighted bimodal distribution (*n* = 3).

Type of Liposomes (Sonication Time)	Peak 1 *		Peak 2 *		PI
	Size (nm)	%	Size (nm)	%	
Chitosan-containing (1 min)	44.9	35	188.7	59	0.357
Carbopol-containing (1 min)	90.9	15	508.6	83	0.456
Carbopol-containing (2 min)	72.0	15	401.6	85	0.517
Plain (1 min)	41.4	13	224.5	86	0.368

* The values are presented as NICOMP distribution, which provided the best fit for the measured data (Fit error <1.5; residual error <10).

**Table 2:** Zeta potential of liposomes. All values represent the mean ± SD (*n* = 3).

Type of Liposomes (Sonication Time)	Zeta Potential (mV)
Chitosan-containing (1 min)	10.6 ± 1.3
Carbopol-containing (1 min)	−4.2 ± 0.4
Carbopol-containing (2 min)	−2.3 ± 0.5
Plain (1 min)	−0.5 ± 0.7

**Table 3:** Antifungal activity of different formulations.

Formulation	*C. albicans* Inhibition
	Chitosan (mg/mL)
Chitosan-containing (MTZ)	0.11–0.22
Chitosan-containing (no drug)	0.11–0.22
Carbopol-containing (MTZ)	No inhibition
Plain (MTZ)	No inhibition
Metronidazole in solution (control)	No inhibition

MTZ: Metronidazole.
